# Genetic determinants of mate recognition in *Brachionus manjavacas *(Rotifera)

**DOI:** 10.1186/1741-7007-7-60

**Published:** 2009-09-09

**Authors:** Terry W Snell, Tonya L Shearer, Hilary A Smith, Julia Kubanek, Kristin E Gribble, David B Mark Welch

**Affiliations:** 1 School of Biology, Georgia Institute of Technology, Atlanta, GA 30332-0230, USA; 2 Josephine Bay Paul Center, Marine Biology Laboratory, Woods Hole, MA 02540, USA

## Abstract

**Background:**

Mate choice is of central importance to most animals, influencing population structure, speciation, and ultimately the survival of a species. Mating behavior of male brachionid rotifers is triggered by the product of a chemosensory gene, a glycoprotein on the body surface of females called the mate recognition pheromone. The mate recognition pheromone has been biochemically characterized, but little was known about the gene(s). We describe the isolation and characterization of the mate recognition pheromone gene through protein purification, N-terminal amino acid sequence determination, identification of the mate recognition pheromone gene from a cDNA library, sequencing, and RNAi knockdown to confirm the functional role of the mate recognition pheromone gene in rotifer mating.

**Results:**

A 29 kD protein capable of eliciting rotifer male circling was isolated by high-performance liquid chromatography. Two transcript types containing the N-terminal sequence were identified in a cDNA library; further characterization by screening a genomic library and by polymerase chain reaction revealed two genes belonging to each type. Each gene begins with a signal peptide region followed by nearly perfect repeats of an 87 to 92 codon motif with no codons between repeats and the final motif prematurely terminated by the stop codon. The two Type A genes contain four and seven repeats and the two Type B genes contain three and five repeats, respectively. Only the Type B gene with three repeats encodes a peptide with a molecular weight of 29 kD. Each repeat of the Type B gene products contains three asparagines as potential sites for N-glycosylation; there are no asparagines in the Type A genes. RNAi with Type A double-stranded RNA did not result in less circling than in the phosphate-buffered saline control, but transfection with Type B double-stranded RNA significantly reduced male circling by 17%. The very low divergence between repeat units, even at synonymous positions, suggests that the repeats are kept nearly identical through a process of concerted evolution. Information-rich molecules like surface glycoproteins are well adapted for chemical communication and aquatic animals may have evolved signaling systems based on these compounds, whereas insects use cuticular hydrocarbons.

**Conclusion:**

Owing to its critical role in mating, the mate recognition pheromone gene will be a useful molecular marker for exploring the mechanisms and rates of selection and the evolution of reproductive isolation and speciation using rotifers as a model system. The phylogenetic variation in the mate recognition pheromone gene can now be studied in conjunction with the large amount of ecological and population genetic data being gathered for the *Brachionus plicatilis *species complex to understand better the evolutionary drivers of cryptic speciation.

## Background

One of the most important decisions in the life of animals is mate choice, which includes discrimination of conspecifics, avoidance of inbreeding, and identification of disease-free, high fitness genotypes. In many animals, pheromones are used to communicate information about the sex, age, and fitness of potential mates [1], and a wide variety of molecules are used to regulate life history transitions [2]. Much is known about mate choice behavior, but little is known about the genes controlling this behavior [3]. Recent work has elucidated some chemosensory genes involved in mate choice and how genetic variation in sensory genes influences mate discrimination. Comparative genomics has shown that genes involved in mate choice typically are highly polymorphic and arise through gene duplication, then are co-opted for mate choice [3]. Reproductive genes often are rapidly evolving and exhibit the signature of positive selection [4].

Brachionid rotifers exhibit mate choice [5-8]. They are capable of both sexual (mixis) and asexual reproduction, where mixis is triggered by a quorum sensing process [9]. If unfertilized, mictic females produce haploid males that are fast swimmers and do not feed [10]. Upon encountering a female, a male exhibits a distinctive mating behavior that consists of tight circling around the female while maintaining contact with his corona and penis. The moment of male-female encounter is critical because this is when a male makes the key decision of whether or not to mate. Male mate recognition is based upon the mate recognition pheromone (MRP), a glycoprotein that is located on the body surface of females [11]. Biochemical properties of the MRP from *Brachionus manjavacas *have been characterized [11-14]. The species *B. manjavacas *is a recently named clade [15] from the *B. plicatilis *species complex [16] and it was formerly known as *B. plicatilis *Russian. MRP can be removed by ethylenediaminetetraacetic acid (EDTA) treatment and transferred to the body surface of conspecific or heterospecific females [17]. Males are thought to detect the MRP signal by contact chemoreception through a receptor in their corona. Males are capable of discriminating conspecifics by geographic origin, sex, and female age [5,7,10,11,18,19], demonstrating a preference for young conspecific females [8,19]. The basis of this choosy mating behavior of males is female MRP signal quality and strength, which in turn determines male mating persistence and circling intensity. Similar male mate choice has been observed in other rotifer species, including *Brachionus calyciflorus *[5,6], *Asplanchna brightwelli *[20], and *Epiphanes senta *[21]. Females also can exhibit mate choice by varying their resistance to male circling [8].

The MRP likely is a critically important molecule in the establishment and maintenance of reproductive isolation among rotifer species [10]. Knowledge of the amount and pattern of variation at both the nucleotide and amino acid levels in natural populations would provide insight into the type and intensity of selection acting on mating barriers. The rate of evolution of reproductive proteins is known to be generally faster than for other proteins [4], but the reason for this is not clear. The evolutionary dynamics of mate recognition genes, their comparative divergence in sympatry and allopatry, and their divergence with local adaptation, remain a mystery. Explaining the abundance of cryptic species in rotifers and how morphologically similar species can coexist and maintain their species integrity also remains a challenge.

A key to answering these questions is the identification and characterization of the MRP gene and developing a hypothesis of its evolution. In this paper, we report on isolation of the MRP protein and determination of its N-terminal sequence. We used the N-terminal sequence to identify from genomic and cDNA a family of genes encoding extracellular matrix proteins, which have various numbers of MRP motif repeats. The predicted protein product of one of these genes, designated MMR-B3, has the biochemical properties expected of the MRP, and we used RNAi to validate the functional role of *MMR-B3 *as the MRP gene in rotifer mating.

## Methods

### Rotifer culture

The strain of *Brachionus manjavacas *[15] was originally collected from the Azov Sea [13] and formerly known as *B. plicatilis *Russian [22]. It is part of the Manjavacas clade as defined by phylogenetic analysis of *COI *and *ITS *genes [7,16,23]. This strain has been cultured continuously in the laboratory since 1983, with periodic collection and storage of resting eggs. Methods for rotifer culture, isolation of males, and hatching of diapausing embryos (resting eggs) followed Snell *et al*. [8].

### Purification and amino acid sequencing of MRP

Approximately 40 to 50 g wet-weight rotifer biomass was filtered from 250 liter mass cultures fed the green alga *Tetraselmis suecica*. Isolation of protein followed the EDTA extraction protocol of Snell and Stelzer [17]. Extracted proteins were separated by SDS-PAGE, following the methods of Snell and Stelzer [17], and visualized with Sypro Orange protein gel stain (Molecular Probes, Eugene, OR, USA) according to the manufacturer's protocol. The detection limit for this stain is about 25 ng. Ion-exchange fractions capable of eliciting male mating reactions eluted from the column at 21 to 23 minutes [17], and were subjected to high-performance liquid chromatography (HPLC) chromatography as described in Snell *et al*. [22]. Edman N-terminal amino acid sequence of the 29 kD protein band from the 17 minute HPLC fraction was determined by the Emory University microsequencing facility from a Western blot of a PAGE gel. Sequencing yielded a 0.7 pmol signal for the 29 kD protein.

### Isolation and sequencing of the gene encoding MRP

The N-terminal amino acid sequence was used to screen an existing *B. manjavacas *cDNA library (Mark Welch *et al*., in prep) using TBLASTN. Two contigs were identified with translated open reading frames containing close matches to the N-terminal sequence of the 29 kD band (see Results). As both contigs contained repeats of a conserved motif we designated these candidate MRP genes MRP Motif Repeat A (*MMR-A*) and B (*MMR-B*). Primers based on the 5'- and 3'-ends of putative MRP transcripts were used in polymerase chain reaction (PCR) of *B. manjavacas *cDNA and genomic DNA (Table 1). To extract genomic DNA or construct cDNA, *B. manjavacas *was collected on a sterile 40 μm Nitex mesh, rinsed with 15 ppt artificial seawater (ASW) followed by deionized water, and pipetted into a 1.5 ml microcentrifuge tube. The rotifers were pelleted by centrifugation and the supernatant was aspirated. Genomic DNA was extracted using the DNeasy Blood and Tissue kit (Qiagen), following the manufacturer's instructions. For cDNA construction, total RNA was extracted with the RNAqueous^®^-Micro kit (Ambion, Austin, TX, USA), and cDNA was synthesized following the FirstChoice^® ^RLM-RACE kit (Ambion), except that mRNA was reverse transcribed with Superscript II™ reverse transcriptase (Invitrogen, Carlsbad, CA, USA) primed with the polyT containing oligonucleotide CDSIII (BD Bioscience, San Jose, CA, USA) to favor full-length transcripts.

**Table 1:** Primers used in the study.

**Gene**	**GenBank Accession Number**	**Primer**	**Primer sequence (5'-3')**
*MMR-A4*	GQ374516	MMRA4F	ATGAAATCAATTTTATGTTCCTSCTG
		
		MMRA4R	TTAATCARAATAAAGAGGAAAAG

*MMR-A7*	GQ374517	MMRA7F	ATGAAATCAATTTTATGTATCCTSCTG
		
		MMRA7R	GTATTTTTTATTTTTGATAAAAATCTG

*MMR-B3*	GQ374518	MMRBF	GTACCAGTYAAGCAAATAGCTGAACC
		
		MMRBR	ATATTTTAAATTTAACCTTGAACC

*MMR-B5*	NA	MMRBF	GTACCAGTYAAGCAAATAGCTGAACC
		
		MMRBR	ATATTTTAAATTTAACCTTGAACC

*MMR *for RNAi (vector-specific)	NA	RNAiF	ACCATGATTACGCCAAGCTCAG
		
		RNAiR	GTTTTCCCAGTCACGACGTTG

*MMR *(vector-specific for double-stranded RNA synthesis)	NA	RNAi-T7F	TAATACGACTCACTATAGGACCATGATTACGCCAAGCTCAG
		
		RNAi-T7R	TAATACGACTCACTATAGGGTTTTCCCAGTCACGACGTTG

GDP-mannose 4,6-dehydratase (MAN)	FJ829249	MANF MANR	GGGGTATGTTTTGTCCCAATC ACCCAGCAGCATATGGTTTC
		
		MANF-T7F MANR-T7R	TAATACGACTCACTATAGGGGGGTATGTTTTGTCCCAATC TAATACGACTCACTATAGGACCCAGCAGCATATGGTTTC

NA = not applicable.

Amplifications for *MMR-A *were performed in 50 μl reactions containing 100 ng cDNA or 500 ng genomic DNA, 0.2 μM each primer (Table 1), 0.4 mM dNTPs, 0.75 M betaine, 2.5 U *Taq *polymerase (Qiagen), and 5 μl ×10 buffer (Qiagen). PCR conditions were 1 minute at 94°C followed by 35 cycles of 15 seconds at 94°C; 30 seconds at *T*_a_; 120 seconds at 72°C followed by 7 minutes at 72°C, where *T*_a _was 45°C, 48°C, 52°C or 55°C. Amplifications for *MMR-B *were performed in 25 μl reactions containing 1× TopTaq Reaction Buffer (Qiagen), 50 ng genomic DNA, 200 μM dNTPs, 0.4 μM each primer, and 1 U TopTaq (Qiagen). PCR conditions were 4 minutes at 94°C, followed by 27 cycles of 30 seconds at 50°C, 20 seconds at 50°C, and 2 minutes at 72°C, followed by 7 minutes at 72°C.

Amplification products were visualized on agarose gels, extracted using a MinElute Gel Extraction Kit (Qiagen), ligated into pCR4-TOPO using the TA-cloning reaction, and transformed into *Escherichia coli *Top10 cells following the supplier's protocol (Invitrogen). Plasmids were extracted from positive clones and sequenced in both directions using standard M13 forward and reverse primers with ABI Big Dye 3.1 chemistry on an ABI 3730 × l Genetic Analyzer. The sequences were edited and assembled using PHRED, CROSSMATCH, and PHRAP[24] as previously described [25] or using Sequencher 4.6 (GeneCodes Corporation).

As two of the candidate MRP genes could not be reliably assembled from forward and reverse reads of the PCR products due to their length and repeat structure, we constructed a *B. manjavacas *fosmid genomic library using standard methods as previously described [26] and probed membranes representing about a 2-fold genome coverage using a ^32^P-labeled portion of MRP candidate gene *MMR-A4 *(4 indicating the number of repetitive motifs within the sequence). A positive fosmid was sub-cloned by shearing the fosmid DNA to about 3 kb fragments by nebulization, repairing the ends by extension with *Taq *polymerase, and ligating the repaired fragments into pCR4-TOPO. Plasmid clones were sequenced as described above. Due to the repetitive nature of the *MRP *candidate genes the sequences were assembled by hand using Sequencher 4.6. To resolve a large repeat region, the 2.1 kb insert of a sub-clone containing the entire region with 100 to 150 base pairs (bp) of flanking DNA was excised from pCR4-TOPO with *Eco*RI (which does not cut in the insert), eluted on an agarose gel and extracted with a MinElute column. The purified insert was partially digested with *Rsa*I, a blunt cutter that cuts once in each repeat (500 ng purified DNA; 0.2 U *Rsa*I; 1× New England Biolabs Buffer 1 for 5 minutes at 37°C). Digestion products were eluted on an agarose gel, extracted with a MinElute column, phosphatased using Shrimp Alkaline Phosphatase (USB, Cleveland, OH, USA) for 60 minutes at 37°C, and cloned into pCR4-TOPO as described above. The insert sizes of 96 clones were checked by digestion with *Eco*RI and 43 were sequenced and assembled using the non-repetitive flanking sequences as guides. This resulted in an unambiguous assembly with 28× average read coverage and no bp disagreements. Sequences were analyzed using the EMBOSS suite [27] and DnaSP [28]. Signal peptides were detected using SIGNALP[29], which predicts the presence of an N-terminal peptide sequence that signals for secretion, and the peptidase I cleavage site that removes the peptide, using hidden Markov models and neural networks based on known signal peptide sequences.

### Synthesis of double-stranded RNA

Plasmids containing *MMR-A *or *MMR-B *genes were used as templates for double-stranded (dsRNA) synthesis. The *MMR-A4 *plasmid insert originated from PCR amplification from fosmid DNA. The MMR-A7 insert was a sub-clone of a fosmid. The *MMR-B3 *insert was derived from a PCR product from genomic DNA. Due to the repetitive motifs in the putative MRP genes, primers were designed to the pCR4-TOPO vector (Table 1) and used to amplify a single product containing *MMR-A7 *or *MMR-B3 *flanked by around 80 vector nucleotides at the 5'- and 3'-ends. GDP-mannose 4,6-dehydratase isoform 1 (referred to as 'MAN' throughout) was amplified from genomic *B. manjavacas *DNA using primers from Snell *et al*. (Snell TW, Shearer TL, Smith HA: Exposure to dsRNA produces RNA interference in *Brachionus manjavacas *(Rotifera). *BMC Genomics*, submitted) (Table 1).

MRP and MAN PCR amplifications were performed in 10 μl volume solutions with 10 to 50 ng plasmid DNA (*MMR-A7 *and *MMR-B3*) or genomic DNA (MAN), 0.2 U *Taq *DNA polymerase and a final concentration of 0.2 mM of each dNTP, 10 mM Tris-HCl (pH 8.3), 50 mM KCl, 0.001% gelatin, 2.5 mM MgCl_2_, and 0.2 μM vector-specific (*MMR-A7 *and *MMR-B3*) or gene-specific primers (Table 1). Thermal cycling conditions consisted of 95°C for 2 minutes followed by 40 cycles of 95°C for 30 seconds, 50°C for 90 seconds, 72°C for 90 seconds. PCR amplicons were gel-extracted using the QIAquick Gel Extraction Kit (Qiagen).

Gel-extracted PCR products were used as template (1 μl) for a subsequent PCR using vector-specific (*MMR-A7 *and *MMR-B3*) or gene-specific (MAN) primers with T7 bacterial promoter sequence at the 5'-end of each primer (Table 1). PCR amplification conditions were the same as above. Products were visualized on a 2% agarose gel, and gel-extracted using a QIAquick Gel Extraction Kit (Qiagen).

dsRNA of the candidate MRP and MAN genes and synthesized by *in vivo *transcription in 33 μl reactions following a modification of Piano *et al*. [30]. Reactions consisted of 15 μl T7 PCR product (about 7 to 9 ng/μl), 1.21 mM NTPs, 4.53 mM DTT, 12.1 mM Tris (pH 7.9), 1.8 mM MgCl_2_, 0.6 mM spermidine, 3.0 mM NaCl, and 90 U T7 RNA polymerase (Promega, Madison, WI, USA), and were incubated at 37°C for 4 hours. The product was ethanol precipitated and purified dsRNA was re-suspended in 5 μl sterile, RNase-free water. To minimize degradation of the dsRNA, RNAi experiments were conducted within 48 hours of dsRNA production.

To confirm that the transcribed dsRNA was from *MMR-A7 *and *MMR-B3*, PCR products from the *MMR-A7 *and *MMR-B3 *PCR using the T7 primers were cloned using a TOPO TA Cloning kit (Invitrogen) and sequenced in both directions using M13 primers (Nevada Genomics, Reno, NV, USA). Sequences were aligned with *MMR-A *and *MMR-B *cDNA sequences using BioEdit v7.0.5 [31], confirming the dsRNA used in RNAi experiments was from the MMR genes (data not shown).

### Transfection protocol

Diapausing embryos were decapsulated and transfected with dsRNA following the double transfection technique of Snell *et al*. (Snell TW, Shearer TL, Smith HA: Exposure to dsRNA produces RNA interference in *Brachionus manjavacas *(Rotifera). *BMC Genomics*, submitted). For the resting egg and the subsequent feeding transfection, 10 to 50 ng dsRNA was resuspended in 5 μl water. Mating bioassays were performed on maternal females approximately 24 hours following the feeding transfection.

### Mating bioassay

Resting egg hatchlings are all females and were collected under a stereomicroscope at 10× magnification using a narrow bore glass micropipette. Males were filtered from a 5 to 7-day-old 200 ml culture using a 68 μm mesh pore size Nitex screen and re-suspended in ASW of the same salinity as the culture (12 to 16 ppt). Only vigorous, fast swimming males (ages unknown) were isolated and mated with the treated females. The mating bioassay was performed by placing seven males and one female into about 50 μl of ASW on a microscope slide printed with 10 spots. Mating behavior was observed for 3 minutes on a video monitor at 10× magnification using a CCD camera. The number of male-female encounters and the number of matings initiated (circlings) by males were recorded using 6 to 12 replicate females for each treatment. The number of encounters and circlings of males with control females, and with females exposed to a variety of HPLC fractions (described below) or dsRNA transfections of *MMR-A7 *and *MMR-B3*, were compared. To determine synergistic effects of *MMR-A7 *and *MMR-B3 *knockdown, a transfection was performed in which *MMR-A7 *and *MMR-B3 *at half dosages were together complexed with the lipofection reagent (Snell TW, Shearer TL, Smith HA: Exposure to dsRNA produces RNA interference in *Brachionus manjavacas *(Rotifera). *BMC Genomics*, submitted), for a total quantity of dsRNA similar to *MMR-A7 *or *MMR-B3 *alone.

A variation on the mating bioassay was used to test the ability of the HPLC fractions to elicit male mating reactions. Rotifer females were exposed to 50 mM EDTA then washed following Snell and Stelzer [17] to strip off their surface glycoproteins, making them receptive to binding of exogenous proteins. Females treated with EDTA were exposed to HPLC protein fractions to test the ability of each fraction to elicit male mating. Exposure of six to eight EDTA-treated females was performed using the protocol for treatment with ion-exchange fractions described in Snell and Stelzer [17]; control females were exposed to EDTA but not treated with protein fractions.

The null hypothesis tested in the mating bioassays was that the frequency of circling is independent of the treatments, which were an HPLC fraction or a dsRNA transfection. For RNAi assays, controls consisted of females transfected with phosphate-buffered saline (PBS) in place of dsRNA; PBS is a reagent in the transfection solution. A one-way analysis of variance (ANOVA) compared circling of control and treated females [32]. The probability of male circling of 6 to 12 replicate females was recorded for each treatment. An ANOVA was performed on raw and arcsine transformed data. Since there was virtually no difference in the ANOVA values, we present results for the untransformed data.

## Results

### HPLC fractions containing the MRP

**Figure 1:**
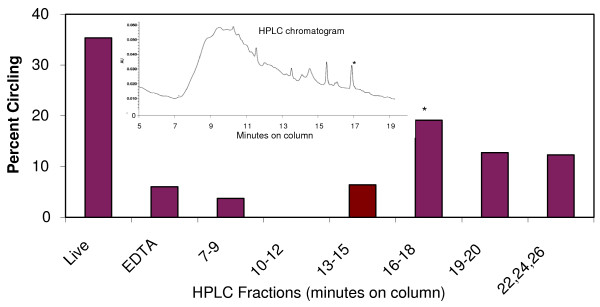
**High performance liquid chromatography purification of mate recognition pheromone from ethylenediaminetetraacetic acid extract**. *x*-axis refers to minutes that the high performance liquid chromatography (HPLC) fractions were retained on the column. Live category is live female rotifers that served as a positive control. Ethylenediaminetetraacetic acid (EDTA) category is rotifer females that had their surface glycoproteins removed by EDTA extraction (negative control). Percent circling on the *y*-axis is from the mating bioassay. The inset is the HPLC chromatogram with retention time in minutes on the *x*-axis and absorbance units at 280 nm on the *y*-axis. Asterisk indicates fraction with significant mating activity.

The HPLC chromatogram revealed six major peaks eluting from the column between 13 and 18 minutes (Figure 1). The fraction most consistently inducing significant male circling eluted slightly before 17 minutes. This fraction produced a 3.2× higher frequency of male circling than the EDTA negative control (Fisher's exact test, *P *= 0.002), and 54% of the circling observed in the live female positive control.

### SDS-PAGE of HPLC fractions

**Figure 2:**
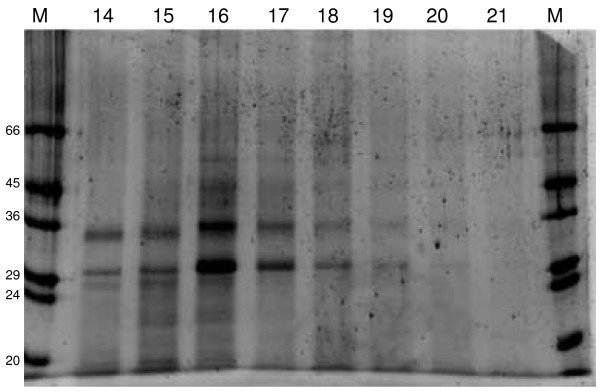
**SDS-PAGE of electrophoretically separated proteins stained with SYPRO Orange**. M indicates the marker protein lane with molecular weights in kD. Numbers across the top refer to high performance liquid chromatography fractions and represent retention times in minutes.

**Figure 3:**
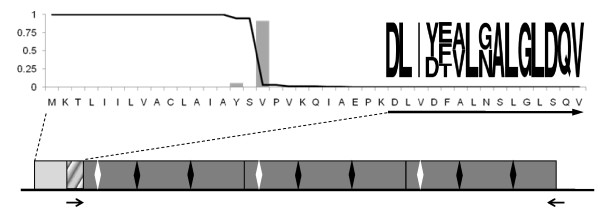
**Properties of *MMR-B3***. The gene transcript (bottom) is composed of an 11 base 5'-end untranslated region (UTR), an 849 base coding region (rectangle), and a 34 base 3'-end UTR. The coding region begins with a 16 codon region encoding a signal peptide (light gray box) followed by 10 additional codons (hashed box) before the first of three repeats of a conserved motif (dark gray boxes). Arginines within each repeat are indicated with diamonds. The first two motifs are 87 codons each and differ at a single synonymous position; the third motif is truncated after codon 83 by a stop codon (TAA) and differs from the second by a single synonymous position. The 3'-end UTR bears no resemblance to the remainder of the motif. The first 41 amino acids of the predicted peptide (top) show the probability of each residue being part of a signal sequence (line) and the probability of each residue being on the carboxy side of the signal peptide cleavage site (histogram). The sequence logo to the right shows the N-terminal sequence of the 29 kD band, scaled to the frequency of each residue. The left arrow (below) shows the PCR primer matching the beginning of the first repeat, which also matches the N-terminal sequence of the 29 kD band. The right arrow represents the reverse primer. The predicted masses of the full-length peptide, the peptide without the signal region, and the peptide beginning with the first base of the first repeat (dark boxes only) are 30.9 kD, 29.2 kD, and 28.1 kD, respectively; the isoelectric points are 4.26, 4.16, and 4.02; and the charges at pH 8.0 are -9.4, -10.1, and -11.1.

The HPLC fractions differed markedly in protein composition (Figure 2). The 14 minute fraction contained three dominant bands with apparent molecular masses of 34, 29, and 26 kD. The 34 kD band also was visible in the 15 to 19 minute fractions, but absent from fractions 20 and 21. Likewise, the 29 kD band was clearly visible in fractions 14 to 19, but absent from fractions 20 and 21. The 26 kD band was clearly visible only in fractions 14 and 15. In all fractions there were a few to several additional faintly visible protein bands. Only the 16 to 18 minute fractions possessed significant ability to elicit male mating behavior, and its most prominent band was 29 kD. N-terminal sequencing was performed on Western blots of two independent samples of the 29 kD band, previously identified as a candidate for the MRP protein [11]. The first sequencing yielded D L I Y/D F/E/T V/A L G/N A L G L D Q V (Figure 3), where the/indicates uncertainty about which amino acid resides at the position. The second sequencing yielded X L I Y F/E V/A L G/N A L.

### Identification and characterization of candidate MRP genes

The *B. manjavacas *cDNA library had two contigs with translated open reading frames containing close matches to the N-terminal sequence of the 29 kD band (Figure 3). The two contigs can be aligned with only minor gaps but differ by more than 50% of aligned nucleotides. As both contigs contained repeats of a conserved motif we designated the two sequences MRP Motif Repeat (MMR) types A and B. The first contig, designated *MMR-A*, appeared to be an incomplete transcript lacking the 5'-end: though sequenced in both directions from four clones none of the clones contained a sequence consistent with a 5'-end untranslated region (UTR) or with the 5'-end signal peptide region discussed below. The assembly of the reads, and thus the true length of the partial transcript, was ambiguous due to the near identity of the motif sequence across repeats. Sequencing a fosmid clone obtained from probing the *B. manjavacas *genomic library with MMR-A revealed two *MMR-A *genes with four and seven repeats, which we designated *MMR-A4 *and *MMR-A7*, respectively, and provided their full coding sequence. Other than differing in the number of repeats of the motif, the coding regions of *MMR-A4 *and *MMR-A7 *are nearly identical, differing at only 2% of aligned nucleotides. Using primers designed to the 5'-end of the coding sequence and to the junction of the 3'-end of the coding sequence and the 3'-end UTR, we amplified *MMR-A4 *from both cDNA and genomic DNA. There was no evidence of additional copies of *MMR-A*. Comparing the genomic and cDNA sequences of the two genes showed no evidence of introns or splicing for either gene.

The contig from the cDNA library representing sequence type *MMR-B *was made up of a single clone sequenced in both directions. As both reads spanned the entire insert, which contained an open reading frame flanked by 5'- and 3'-ends UTRs, the reads could be assembled unambiguously despite the presence of three repeats. We designed primers to the 5'- and 3'-ends of this transcript, *MMR-B3*, and used them to amplify cDNA and genomic DNA. The only two amplification products were *MMR-B3 *and a closely similar *MMR-B *gene with five repeats of the motif, *MMR-B5*. Comparing the genomic and cDNA sequences of the two genes showed no evidence of introns or splicing for either gene.

The conceptual translations of the MMR genes all share several features: each begins with a signal peptide region followed by nearly perfect repeats of a motif of 92 amino acids (*MMR-A*) or 87 amino acids (*MMR-B*); the terminator interrupts the final motif after amino acid 85 (*MMR-A*) or 81 (*MMR-B*) and there are no amino acids between repeats of the motif (Figure 3). The motif is 55% to 60% non-polar with a net negative charge, and is predicted to be composed of a series of alpha helices, producing a generally globular protein. The region corresponding to the N-terminal sequence obtained from the 29 kD band begins at the third amino acid of the first motif (*MMR-A*) or at the first amino acid of the first motif (*MMR-B*). The *MMR-A *genes lack codons for asparagine, and thus MMR-A protein products are unlikely to be N-glycosylated. Each motif in the product of the *MMR-B *genes contains three asparagines as potential sites for N-glycosylation, although they do not occur in the series N-X-T, which generally marks N-glycosylated asparagines. Two of the three asparagines are predicted to be on the hydrophilic sides of alpha helices, and therefore potentially exposed. None of the processed MMR peptides has significant similarity to any sequence in GenBank/EMBL/DDJ databases as judged by BLASTX and BLASTN searches, or to known protein motifs as judged by HMMPFAM searches of PFAM databases. After cleavage of the signal peptide the molecular weights of the peptides produced by MMR-A4, -A7, -B3, and -B5 are predicted to be 38.4 kD, 69.2 kD, 29.2 kD, and 48.2 kD respectively; each peptide is predicted to be negatively charged at pH 8.

### MRP RNAi knockdown

**Figure 4:**
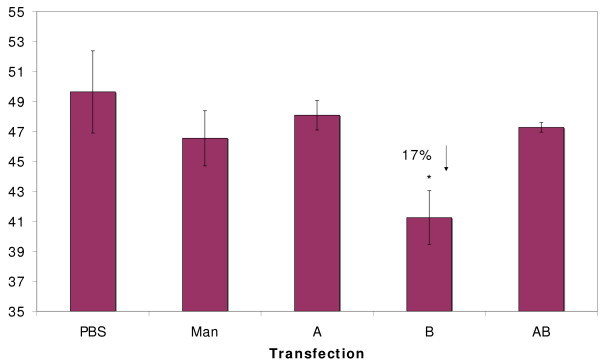
**RNAi knockdown of *MMR-A7 *and *MMR-B3 *genes and comparison of their effects on male mating behavior**. All transfections are compared with a negative control transfected with phosphate-buffered saline instead of double-stranded RNA (dsRNA). AB was transfected with a half dose of *MMR-A7 *plus a half dose of *MMR-B3*. MAN was transfected with dsRNA from the GDP-mannose 4,6-dehydratase gene. Percent circling is the number of circling behaviors performed by males divided by the number of male-female encounters. Asterisks indicate significant reductions in male circling; percentages with downward arrows indicate the magnitude of the reduction. Vertical lines on top of each column represent standard errors.

Transfection of females with dsRNA for *MMR-A7 *did not result in less circling than in the PBS control (Figure 4). In contrast, transfection with MMR-B3 reduced male circling by 17%, which is significantly less than the PBS control (one-way ANOVA, *F *= 5.74, degrees of freedom = 9, *P *= 0.043). Neither the combined transfection with *MMR-A7 *and *MMR-B3 *dsRNA, nor the GDP-mannose 4,6-dehydratase (MAN) dsRNA had significant effects on rotifer mating.

## Discussion

### Characteristics of MRP protein

This study is the culmination of extensive efforts to identify the MRP gene, beginning with studies of the role of glycoproteins as mating signals in rotifers. Nearly two decades ago the biochemical properties of surface glycoproteins involved in rotifer mate recognition were probed in binding studies with several fluorescently labeled lectins, which bind the carbohydrate moiety of the MRP protein [12,33]. When these lectins were bound, females elicited significantly fewer mating responses from males. Cleavage of surface proteins by proteinase K also rendered females significantly less attractive to males [34], as did cleavage of N-linked oligosaccharides by the glycohydrolase N-glycanase [11]. Treatment of *B. manjavacas *females with the detergent CHAPS and EDTA removed surface proteins and eliminated the male mating response [12,17]. In the present study, binding of EDTA-extractable proteins to an anion exchange column suggests that the proteins possess a net negative charge at pH 8.0. Adsorption of the surface glycoproteins to a C3 reversed-phase HPLC column, eluting in 64% to 72% acetonitrile, further suggested the proteins contain hydrophobic domains.

While a putative MRP protein had been previously isolated, our study is the first to report its gene sequence and confirm its function. Snell *et al*. [11] described a 29 kD rotifer surface glycoprotein that likely represented the MRP. Yet, the amino acid sequence could not be determined from the small quantity of protein that had been purified, and there is no conclusive way to determine whether the glycoprotein described is identical to the MRP protein reported here. Nonetheless, in the current study, the HPLC fractions of EDTA-extractable proteins capable of eliciting male mating behavior had only two prominent proteins with 34 and 29 kD apparent mass. Given the earlier identification of the putative 29 kD MRP, here we focused on the 29 kD protein as the most likely candidate for the MRP. N-terminal amino acid sequencing of the 29 kD protein yielded enough sequence to perform a meaningful search of a rotifer cDNA library. Reduction of mating from RNAi knockdown of expression of the MMR-B3 gene as reported here validates the 29 kD protein as the MRP, and that this protein is critical for *B. manjavacas *mate recognition.

### Characteristics of the MRP gene

Using the N-terminal sequence of the 29 kD protein, we identified two contigs from a cDNA library of *B. manjavacas *as candidates for the MRP gene. PCR of genomic DNA and cDNA, as well as sequencing a fosmid from a genomic library of *B. manjavacas *containing two candidate genes, revealed that the N-terminal sequence is a marker for a family of related genes. This gene family has no recognizable homology to sequences in public databases or to the protein patterns of PFAM but is characterized by a peptide signal sequence region followed by repeats of a motif that contains the N-terminal sequence at or near its 5'-end. There is a short spacer region between the signal peptide and the repeats but the repeats follow each other head-to-tail without interruption. The final motif is invariably truncated by four to seven codons. The presence of a signal peptide implies that the peptides are secreted via the endoplasmic reticulum (ER), consistent with being part of the extracellular matrix on the surface of the rotifer, and the negative charge and general hydrophobicity is consistent with previous biochemical studies of the MRP protein. The *MMR-A *and *MMR*-*B *genes differ at about 50% of aligned nucleotides (exact homology of the repeats cannot be reliably assigned), but the repeats of a motif within a gene are identical or differ by only a few nucleotides.

Data suggest *MMR-B3 *encodes a functional MRP protein. Despite considerable effort we have not identified a *MMR-A *type gene that would produce a 29 kD peptide. Also, although the MMR-A motif contains a single potential O-glycosylation site, it does not contain arginine, so is unlikely to be N-glycosylated. Finally, RNAi with the MMR-A motif did not decrease male mating behavior. In contrast, one of the *MMR-B *type genes, *MMR-B3*, is predicted to produce a 29 kD product, the MMR-B motif does contain arginines, which are predicted to be on the exposed surface of the protein. Finally, RNAi with the *MMR-B3 *gene significantly reduces mating behavior. Thus, we conclude that MMR-B3 encodes the 29 kD peptide originally identified as the MRP. We hypothesize that after cleavage of the signal region upon entry into the ER the peptide is further modified by the removal of the remaining residues before the first motif, consistent with N-terminal sequencing of the 29 kD band. Also, one or more arginines per motif may be N-glycosylated or another form of oligosaccharide moiety that mimics N-glycosylation in biochemical assays may be added.

That the MRP gene (*MMR-B3*) has no significant similarity to any deposited sequence may not be surprising, as at least 20% of the brachionid transcriptome appears to lack obvious homologs in sequence databases [24], and there is in general very little knowledge of the genes encoding extracellular proteins in non-ecdysozoan protostome phyla. The very low divergence between repeat units, even at synonymous positions, strongly suggests that the units are kept nearly identical through a process of concerted evolution analogous to the preservation of sequence homogeneity in rRNA clusters. Concerted evolution of repeats in turn suggests a mechanism by which a single non-synonymous mutation could rapidly spread to be present in multiple repeats, effecting a major change in the structure of the protein. Such saltational change of a protein involved in mate choice could lead to rapid speciation.

### Comparison of the MRP to contact mate recognition pheromones of other animals

The critical role of a single gene in mate recognition of rotifers makes their signaling pathway for mate recognition particularly useful for studying the molecular mechanisms and evolution of mate recognition, and the role of mating genes in speciation. In contrast, in many other animals a variety of factors are influential, and thus assigning functional roles of genes is difficult. In *B. manjavacas*, glycoproteins from the female are necessary and sufficient to elicit male circling [17]. Chemosensory mate recognition genes also have been described in other animal species [3]. Perhaps most similar to rotifers is the mate recognition system of the fruit fly *Drosophila*. Males evaluate the approximately 20 cuticular hydrocarbons (CH) of females by contact chemoreception to discriminate mates and prefer to mate with same-strain females [35]. Olfactory receptors on the antennae and gustatory receptors on the tarsa and proboscis of males detect immobilized surface heptacosadienes on females of 20 to 40 carbons in chain length [36]. The neurobiological basis of mating decisions in *Drosophila *also is beginning to be revealed [37]. It is now understood that the quantity and blends of various CHs contain information about species and geographic population and males use this information to determine whether or not to mate. Owing to the large number of CHs involved in mate recognition, the genetic basis of mating phenotypes is complex [38] and only a few genes have been characterized [36]. In addition, temperature, food, and social experience can influence CH profiles. This has made it difficult to relate CHs directly to evolutionary processes like sexual selection and to determine explicitly their role in speciation. In contrast, in *B. manjavacas *the MRP is a glycoprotein immobilized on the surfaces of females [11]. It is encoded by a single gene and the MRP phenotype apparently is subject to little environmental modification. The MRP therefore should be an excellent molecular marker for investigating selection and speciation processes in monogonont rotifers.

In comparison with other invertebrates, specifically insects, the composition of the MRP protein provides an indication of how differing selection pressures imposed by terrestrial versus aquatic habitats may have influenced evolution of mating systems. The use of CHs as recognition pheromones by insects is a general theme not limited to *Drosophila *[39-41]. This channel of communication is not surprising since CHs are the most prominent molecules on external surfaces of insects [42]. CHs play a central role in minimizing desiccation [43], and thus their evolution may have resulted from strong selection for desiccation resistance that accompanied the colonization of land. The ability to decipher the information encoded in the molecular composition of cuticles is clearly a useful trait and eventually became the basis of mate recognition in many insects. Aquatic animals have very different cuticles since most have not experienced strong selection for desiccation resistance. For example, Alarie *et al*. [44] found substantial differences between CH profiles of an aquatic beetle compared with its terrestrial counterpart. We suggest that information-rich molecules like surface glycoproteins are well adapted for chemical communication and that aquatic animals may have evolved signaling systems based on these compounds. In this sense, the surfaces of aquatic animals are more like those of individual mammalian cells, which typically are highly decorated with information-rich glycoproteins (see, for example, Gahmberg and Tolvanen [45]). Glycoproteins and their oligosaccharide moieties have been implicated in a wide variety of cell-cell and cell-matrix recognition events [45,46]. By providing evidence of a surface glycoprotein in brachionid rotifers involved in mate recognition of conspecifics, we provide one example of how such compounds are involved in chemical communication in aquatic environments.

Like rotifers, some copepods also use surface glycoproteins to make mating decisions [47]. Mate-guarding in the harpacticoid copepod *Tigriopus japonicus *can be inhibited by binding lectins to surface glycoproteins of females [48,49]. Before males initiate guarding behavior or transfer spermatophores, stroking behavior is engaged in which the male probes the female body surface, presumably in search of chemical cues. The carbohydrate moiety of glycoproteins on the female are thought to be detected by protein receptors on antennules of the male, enabling identification of appropriate partners for mating. Purification of female surface proteins revealed a 70 kD glycoprotein, with significant similarity to the sequence of α_2_-macroglobulin [49,50].

## Conclusion

Here we have isolated the rotifer MRP, identified *MMR-B3 *as the MRP gene, and demonstrated its essential role in male mate recognition by functional assays with RNAi. Our study provides an initial synthesis of behavioral, biochemical, and molecular assays to characterize the mating system of brachionid rotifers. This system may be an important model for comparative studies of mating signaling pathways in aquatic and terrestrial animals. The phylogenetic variation in the MRP gene can now be studied in conjunction with the large amount of ecological and population genetic data being gathered for the *Brachionus plicatilis *species complex to understand better the evolutionary drivers of cryptic speciation. Characterization of the MRP protein will facilitate identification and isolation of the second key element in the rotifer mate recognition signaling system, the male MRP receptor. Males have a variety of receptors in their coronas, and we are investigating a gene in a family of C-type lectins with putative roles in cell adhesion and pathogen recognition [51] as a potential target.

## Abbreviations

ANOVA: analysis of variance; ASW: artificial seawater; bp: base pair; CH: cuticular hydrocarbons; dsRNA: double-stranded RNA; EDTA: ethylenediaminetetraacetic acid; ER: endoplasmic reticulum; HPLC: high performance liquid chromatography; MRP: mate recognition pheromone; PBS: phosphate-buffered saline; PCR: polymerase chain reaction; UTR: untranslated region.

## Authors' contributions

TWS isolated the MRP protein, performed the N-terminal sequencing, the RNAi knockdown experiments, and drafted the manuscript. TLS developed primers, performed PCRs, and the *in vitro *synthesis of dsRNA used for RNAi. HAS developed the dsRNA *in vitro *synthesis protocol. JK assisted in the HPLC and protein purification. KEG screened the cDNA library, identified candidate MRP genes, and sequenced the amplicons. DBMW analyzed the sequences and determined the structure of the MRP genes. All authors edited the manuscript and approve of its final content.
